# The lung-gut-brain axis in non-small-cell lung cancer: a narrative review with a systematic search of three signaling pathways

**DOI:** 10.3389/fimmu.2026.1932225

**Published:** 2026-09-11

**Authors:** Yijun Fan, Lili Du, Qiuping Luo, Tingrui Mei, Aier Zhou, Hangyu Liu, Muhammad Quais Khan, Faxian Gao, Jingsi Dong

**Affiliations:** 1 West China School of Medicine, Sichuan University, Chengdu, China; 2 Department of Endocrinology and Metabolism, The Fourth Affiliated Hospital of Harbin Medical University, Harbin, China; 3 Out-patient Department, West China Hospital, Sichuan University, Chengdu, China; 4 Department of Thoracic Surgery, West China Hospital, Sichuan University, Chengdu, China; 5 Lung Cancer Center/Lung Cancer Institute, West China Hospital, Sichuan University, Chengdu, China

**Keywords:** gut microbiota, metabolic-endocrine axis, neuroimmune integration, non-small-cell lung cancer, soluble metabolites

## Abstract

**Background:**

The gut-lung axis, the long-range communication between the intestinal microbiota and the lungs, has been shown to influence immunotherapy responses in non-small-cell lung cancer (NSCLC). Existing reviews, however, focus almost exclusively on the chemical signaling dimension of microbial metabolites. The relay function of enteroendocrine hormones and the integrative role of the nervous system have yet to be incorporated into a unified framework.

**Methods:**

We searched seven databases (PubMed, EMBASE, Web of Science, Cochrane Library, CNKI, Wanfang, and VIP) from inception to June 2026 for original studies and reviews addressing gut microbiota and lung-gut-brain axis mechanisms in the context of NSCLC or small-cell lung cancer. Two authors independently performed study selection, data extraction, and thematic classification. A narrative synthesis was conducted, with evidence organized along three signaling axes: soluble metabolites, the metabolic-endocrine axis, and neuroimmune integration. As a narrative review with a systematic search, this study was registered on PROSPERO (CRD420261425826), while formal risk-of-bias assessment and GRADE evaluation were not performed.

**Results:**

A total of 168 studies was included (only a subset of these included studies cited in the main text). The volume of evidence across the three axes formed a pronounced gradient. Soluble metabolites (short-chain fatty acids, tryptophan metabolites, and bile acids) have been cross-validated in clinical cohorts, animal models, and *in vitro* experiments; fecal microbiota transplantation and prebiotic strategies have entered clinical trials. The metabolic-endocrine axis rests on a solid foundation in normal physiology, but the key bridging nodes in the NSCLC disease context remain untested. Evidence for the neuroimmune integration axis is the sparsest: the only support for a direct transport function of the vagus nerve comes from a non-peer-reviewed preprint in a non-cancer model, and the full neuroendocrine signal transduction chain remains hypothetical. To address this gap, we outline a pathway model annotated with evidence levels at each node.

**Conclusions:**

Research on the lung-gut-brain axis is heavily concentrated in the chemical dimension. The mechanistic gaps in the endocrine and neural dimensions represent the principal barrier for the field’s transition from correlational description to causal mechanism studies.

## 1 Introduction

Non-small-cell lung cancer (NSCLC) and small-cell lung cancer (SCLC) are the two main histological types of lung cancer. NSCLC accounts for roughly 85% of cases and remains the leading cause of cancer death worldwide. According to GLOBOCAN 2022, lung cancer caused approximately 2.5 million new cases and 1.8 million deaths annually (1). Over the past decade, immune checkpoint inhibitors (ICIs) have reshaped the treatment field, yet only 20-30% of patients derive durable clinical benefit; most face primary or acquired resistance (2, 3) This therapeutic bottleneck has shifted attention from tumor-cell-intrinsic driver mutations toward the host’s systemic immune and metabolic state: tumor progression is driven by oncogenic mutations and by immune and metabolic signals originating in distant organs. In this context, the gut microbiota has moved from being seen as a local ecology of the digestive tract to being recognized as a central node in systemic immune regulation (2, 3).

Microbiota-derived extracellular vesicles can carry nucleic acids, proteins, and metabolites that signal to distal organs (4). Clinical trials combining fecal microbiota transplantation (FMT) with immunotherapy, including an FMT rechallenge study in advanced NSCLC and the phase 2 FMT-LUMINate trial, have further validated the clinical feasibility of microbiome-directed interventions (5, 6). A growing body of evidence indicates that the composition and function of the gut microbiota can substantially influence NSCLC initiation, progression, and immunotherapy response (7, 8).

The gut-lung axis, however, is not a single pathway. It is a complex network comprising at least four parallel signaling dimensions: soluble microbial metabolites, immune cell trafficking, inflammatory cytokine signaling, and direct microbial translocation. Among these, the metabolite pathway has the best-evidenced causal chain. Short-chain fatty acids (SCFAs), bile acids, and tryptophan metabolites are produced by the microbiota, enter the portal circulation, and activate immune receptors in the lung. The evidence spans molecular mechanisms (9–11), reverse-genetic validation (10), and pharmacological tractability (12), and metabolites can be precisely quantified and pharmacologically modulated, offering a clear translational path (13). Yet the metabolite pathway covers only the direct chemical dialog between the gut and the lung. When the central nervous system (CNS) is brought into view, the lung and the gut are also connected to it through endocrine and neural relays, giving rise to the organizing concept of the lung-gut-brain axis. The continuum from microbial homeostasis to systemic pathogenesis, encompassing neuropsychiatric disorders, metabolic diseases, and cancer, provides a unifying theoretical framework for this three-way interaction (14). Within this framework, two additional dimensions can be identified. The first is the metabolic-endocrine axis: enteroendocrine cells (EECs) convert luminal chemical signals into hormonal pulses; glucagon-like peptide-1 (GLP-1), peptide YY (PYY), and 5-hydroxytryptamine (5-HT) reach distal target tissues through the systemic circulation. Cancer cachexia is the phenotypic anchor with the strongest body of evidence (15, 16), but whether specific hormones such as GLP-1 and insulin-like growth factor-1 (IGF-1) mediate effects within the NSCLC pulmonary immune microenvironment has not been directly tested (17). The second is the neuroimmune integration pathway: vagal afferents, the hypothalamic-pituitary-adrenal (HPA) axis, and neuroinflammation form a potential relay circuit, but in NSCLC only associational evidence exists and the causal chain remains unverified (18). Of note, reverse regulation from lung to gut currently has only indirect evidence and is therefore not developed as a separate axis here.

This review centers on NSCLC while drawing on evidence from SCLC and other tumor types for supplementary support. In content organization, it departs from existing correlational summaries by reorganizing the included literature into a causal network along three mechanistic pathways, identifying potential intervention targets within a unified lung-gut-brain axis framework, and grading the research maturity of each pathway to guide future experimental validation and clinical translation.

## 2 Search methods and results

### 2.1 Search strategy

We searched the following Chinese and English databases: PubMed, EMBASE, Web of Science, Cochrane Library, CNKI, Wanfang Data, and VIP. The search covered all records from database inception to June 2026. Languages were restricted to English and Chinese. Reference lists of included studies were hand-searched to capture additional relevant records.

### 2.2 Search query design

Eight complementary search strategies were designed around the three signaling pathways of the lung-gut-brain axis in lung cancer, each capturing literature from a different dimension. Queries were built using PubMed syntax as the baseline and adapted to the syntax of each database. Corresponding Chinese search terms were used in Chinese databases. The 1,215 records reported in Section 1.5 represent the combined total retrieved across all seven databases (the sum of the hits returned by the eight strategies after database-specific adaptation). The complete adapted search strings for all seven databases and the per-database hit counts are provided in **Appendix 1**.

### 2.3 Inclusion and exclusion criteria

#### 2.3.1 Inclusion criteria

Studies were included if they met all of the following: (1) addressed the role of gut microbiota, the gut-lung axis, the gut-brain axis, or the lung-gut-brain axis in the context of NSCLC or SCLC; (2) reported on at least one of the following mechanistic domains: neuroimmune signaling (vagal afferents, HPA axis, neuroinflammation, chemotherapy-related cognitive impairment), neuroendocrine regulation (GLP-1, 5-HT, enteroendocrine hormones, insulin signaling), or soluble microbial metabolites (SCFAs, tryptophan metabolites, bile acids, lipopolysaccharide, trimethylamine N-oxide (TMAO)); and (3) were published as original research articles, reviews, or systematic reviews/meta-analyses in English or Chinese.

#### 2.3.2 Exclusion criteria

Studies were excluded if they met any of the following: (1) focused exclusively on cancers other than lung cancer without providing direct comparative relevance to lung cancer; (2) discussed the gut microbiome or gut-brain axis in a non-oncology context; (3) were published as editorials, commentaries, conference abstracts, errata, or corrections lacking substantive data; or (4) lacked mechanistic content in any of the three target domains.

### 2.4 Study selection

Records retrieved from each database were imported into reference management software. Duplicates were removed automatically and verified manually. Two authors independently performed study selection. Titles and abstracts were first screened to exclude records clearly unrelated to the gut-lung axis or microbiome-lung cancer. Full texts of the retained records were then reviewed against the inclusion and exclusion criteria. Disagreements were resolved by discussion; when consensus could not be reached, the corresponding author adjudicated.

### 2.5 Selection results

The search and study selection process was documented following the Preferred Reporting Items for Systematic Reviews and Meta-Analyses (PRISMA) guidelines (19) and the PRISMA-S extension for search reporting; the flow of records is shown in the PRISMA diagram (**Figure 1**). As this manuscript is a narrative review with a systematic search rather than a systematic review in the methodological sense (Section 1.7), the full PRISMA 2020 checklist does not apply. The search strategies are provided in **Appendix 1**. The initial search across the seven databases yielded 1,215 records. After removing 295 duplicates, 920 unique records entered title and abstract screening. Screening excluded 216 non-lung-cancer studies without lung cancer controls, 147 gut/lung/brain axis studies without a tumor context, 98 editorials/conference abstracts/errata without complete data, and 62 studies lacking core mechanistic content, for a total of 523 exclusions. The remaining 397 records were sought for full-text retrieval; none were inaccessible. Full-text eligibility assessment excluded 94 studies limited to other tumor types without lung cancer controls, 61 gut-lung/gut-brain axis studies in non-oncology settings, 43 commentaries and abstracts without valid research data, and 41 studies missing core mechanistic pathways, for a total of 239 exclusions. The primary search yielded 158 eligible studies. With 10 additional eligible studies identified through supplementary searching, a total of 168 studies were included.

**Figure 1:**
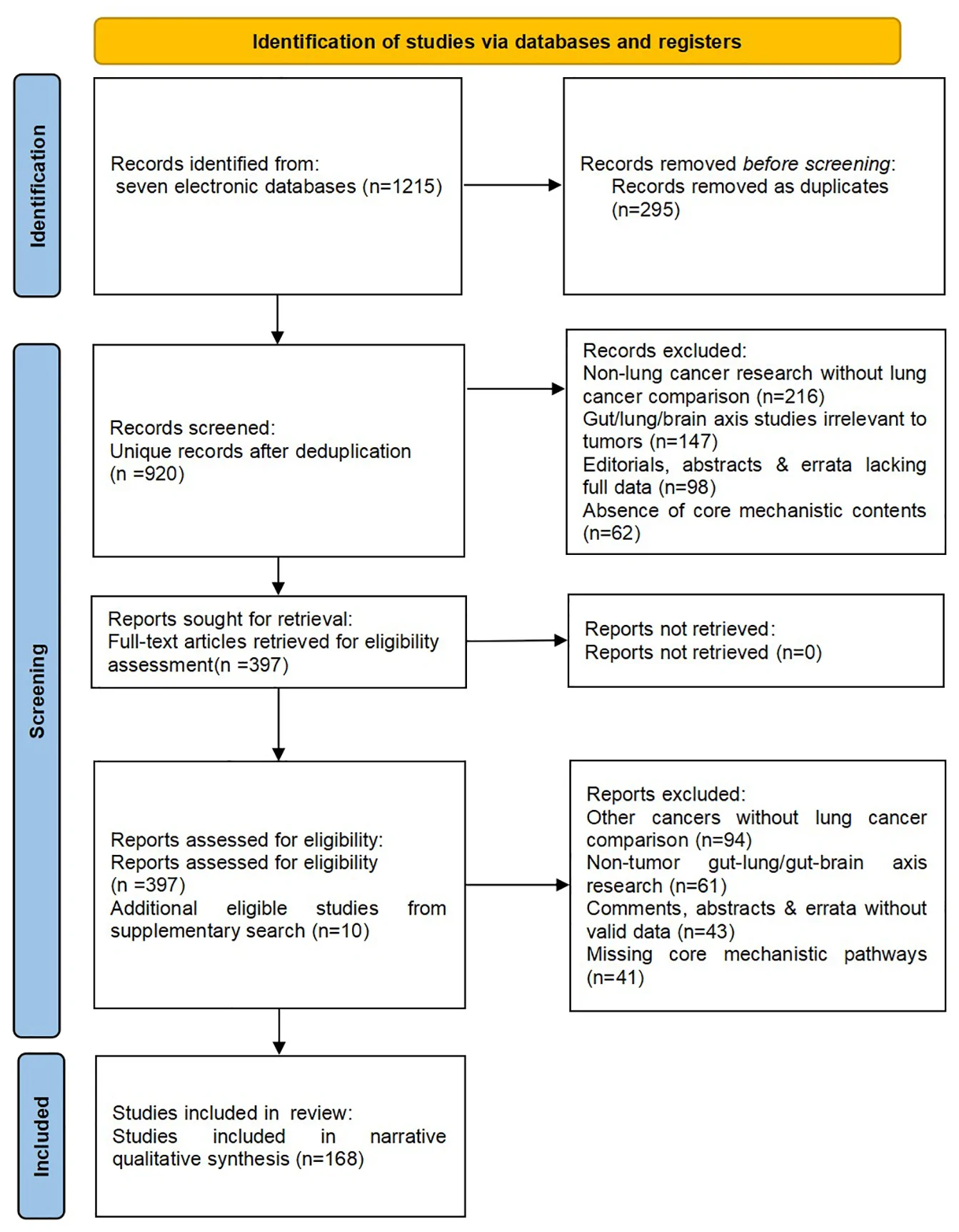
PRISMA flow diagram of study selection. This study followed the PRISMA guidelines for systematic reviews. Systematic searches of seven databases yielded an initial 1,215 records. After deduplication, 920 unique records underwent title and abstract screening; 523 ineligible records were excluded. Full-text assessment of 397 records excluded a further 239 ineligible studies. The primary search yielded 158 eligible studies; with 10 additional studies from supplementary searches, 168 studies were included in the narrative synthesis.

### 2.6 Data extraction

Two authors independently extracted data using a pre-designed standardised extraction form. Extracted items were cross-checked, and discrepancies were resolved by consulting the original article and discussion. Extraction covered: first author, publication year, journal, study type (original research/review/systematic review or meta-analysis), study design, sample size, cancer type (NSCLC/SCLC/pan-cancer), thematic domain (neuroimmune integration/metabolic-endocrine axis/soluble metabolites), main objective, and key findings. The full data-extraction table for all 168 included studies is provided as **Appendix 2**.

### 2.7 Quality assessment

We did not apply formal quality scales or risk-of-bias tools to score each of the 168 studies individually, and the formal GRADE framework was not applied, for three reasons. First, this manuscript is a narrative review with a systematic search, not a systematic review in the methodological sense: its aim is to map and grade the maturity of mechanistic evidence along three signaling axes, not to pool effect estimates for a clinical question. Second, the included studies span highly heterogeneous designs (clinical cohorts, animal models, *in vitro* experiments, and reviews), for which no single quality instrument is appropriate. Third, consistent with this framing, formal quality instruments were not applied; These choices are acknowledged as limitations (Section 3.2). To allow readers to appraise each included study independently, the full data-extraction table is provided as **Appendix 2**.

### 2.8 Data synthesis

Owing to substantial heterogeneity in study designs, interventions, and outcome measures, quantitative meta-analysis was not performed; instead, the present review follows the Synthesis Without Meta-analysis (SWiM) reporting guideline (20). The sources of heterogeneity that precluded quantitative pooling were clinical, methodological, and statistical. Clinically, the included studies span different cancer types (NSCLC, SCLC, and pan-cancer cohorts), treatment contexts (immunotherapy, chemotherapy, and their combinations), and disease stages. Methodologically, they comprise highly diverse designs — clinical observational studies, clinical trials, animal models, *in vitro* experiments, and reviews — with heterogeneous outcome definitions and measurement platforms (e.g., 16S rRNA sequencing, shotgun metagenomics, targeted metabolomics). Statistically, few studies reported directly comparable effect estimates for the same outcome, so effect sizes could not be pooled without substantial distortion. A narrative synthesis was used, organized along the three mechanistic signaling pathways of the lung–gut–brain axis (neuroimmune integration, metabolic–endocrine axis, and soluble metabolites), with literature within each pathway presented by evidence strength. The formal GRADE framework was not applied, as it is primarily designed for quantitative meta-analyses rather than narrative synthesis. The heterogeneity of evidence quality across study types should also be noted: the soluble-metabolites axis is supported predominantly by clinical cohorts with corroborating animal and *in vitro* experiments, whereas the metabolic–endocrine and neuroimmune axes rely more heavily on reviews, normal-physiology studies, and cross-cancer inference. This heterogeneity is reflected in the node-level evidence grading of the pathway model (**Figure 2**) and in the correspondingly cautious wording of the conclusions for the endocrine and neuroimmune dimensions. A quality summary table characterizing the composition of the evidence base is provided in **Appendix 3**.

**Figure 2:**
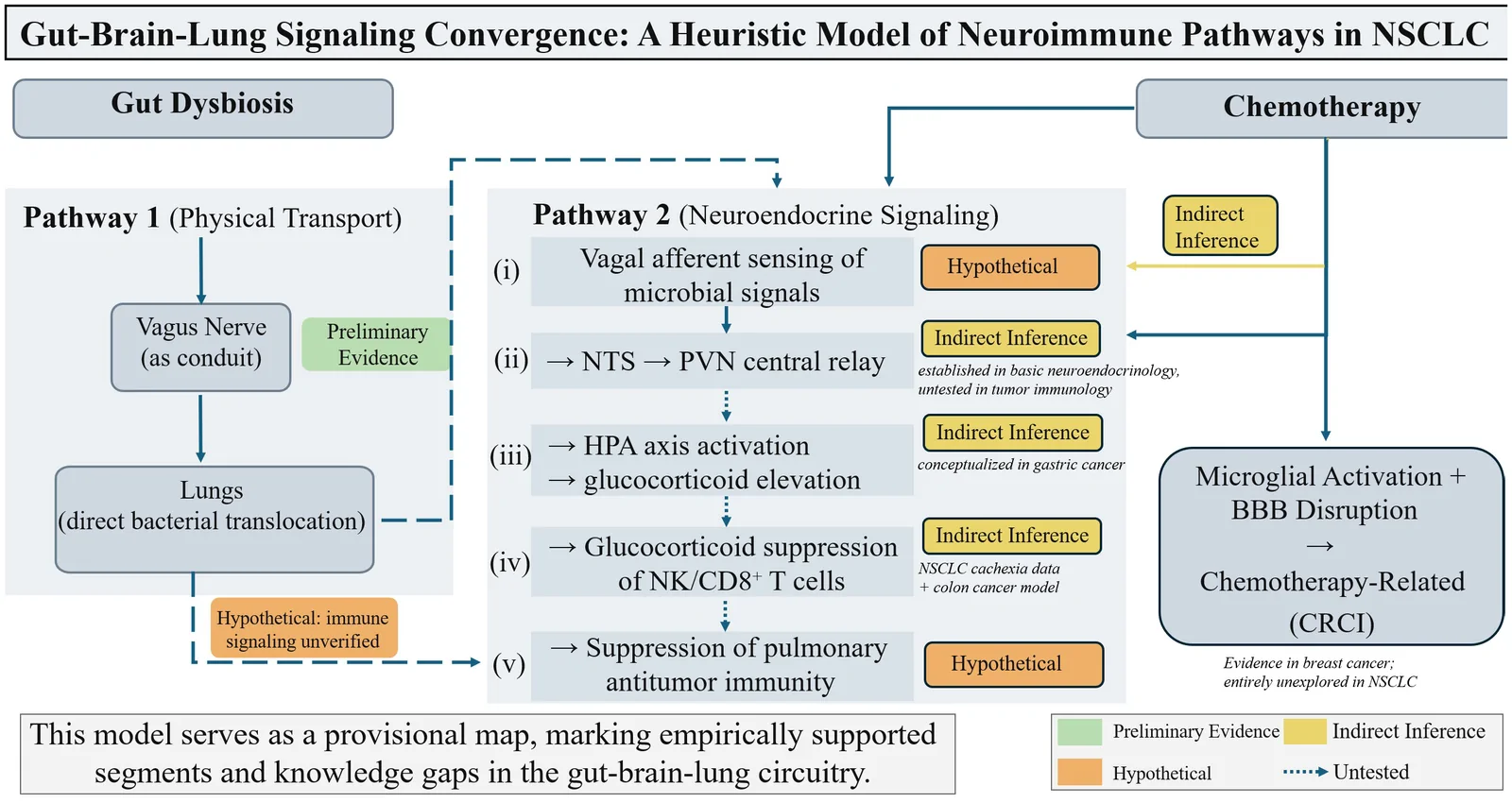
Hypothesis-driven pathway model of the lung-gut-brain axis in NSCLC with node-level evidence annotation. The model annotates the evidence level at each signaling node of the gut-brain-lung axis and covers two core pathways and one chemotherapy-exacerbated branch. Pathway 1 (physical transport): Following gut dysbiosis, viable bacteria can translocate directly to the lungs along the vagus nerve as a physical conduit. This pathway is supported only by preliminary evidence from a single non-peer-reviewed preprint in a non-cancer (interstitial lung disease) mouse model; whether it operates in NSCLC, and whether translocated bacteria modulate pulmonary antitumor immunity, remains hypothetical. Pathway 2 (neuroendocrine signal transduction): Gut microbial signals are sensed by vagal afferent fibers and relayed centrally through the NTS to the paraventricular nucleus (PVN), activating the HPA axis. Elevated glucocorticoids suppress NK and CD8+ T-cell function, ultimately weakening pulmonary antitumor immunity. Most nodes in this pathway are based on indirect inference from other fields; the complete chain has not been validated in NSCLC. The chemotherapy-exacerbated branch converges onto both pathways through dual mechanisms: chemotherapy-induced dysbiosis increases viable bacterial translocation along the vagus nerve (converging on Pathway 1), and chemotherapy-induced systemic inflammation further amplifies HPA axis activation (converging on Pathway 2). In addition, chemotherapy-induced systemic inflammation independently drives microglial activation and BBB disruption, mediating chemotherapy-related cognitive impairment (CRCI). Direct experimental evidence for this branch currently comes primarily from breast cancer studies; it awaits complete validation in NSCLC models.

### 2.9 Thematic classification

Two authors independently classified the 168 included studies into the three mechanistic themes (a single study could be assigned to multiple themes). Disagreements were resolved by discussion, with the corresponding author adjudicating when consensus could not be reached. Classification results are shown in **Table 1**.It is important to note that the thematic classification reflects the breadth of coverage (whether a study contained any content relevant to a theme), not the depth or maturity of the evidence, which is graded separately at the node level (Section 3.1, **Figure 2**). Many of the 153 studies classified under neuroimmune integration address the theme only tangentially (for example, chemotherapy-related cognitive impairment studies performed in other cancer types); a large number of themes-assigned studies therefore does not imply strong direct evidence.

**Table 1:** Distribution of literature across three thematic categories.

Thematic category	No. of studies	Core content
Neuroimmune Integration	153	Vagal afferent signaling, HPA axis activation, neuroinflammation, chemotherapy-related cognitive impairment
Metabolic–Endocrine Axis	105	GLP-1/gut–pancreas–lung axis, 5-HT pro-tumor signaling, insulin–IGF-1 and cancer cachexia
Soluble Metabolites	138	Context-dependent SCFA effects, tryptophan/kynurenine metabolic bifurcation, TMAO, bile acids

Studies may be assigned to multiple themes. Counts represent the breadth of thematic coverage (any content relevant to a given theme), rather than the maturity of available evidence.

### 2.10 Characteristics of included studies

All 168 included studies were published in English: 52 were reviews and other non-original publication types, and 116 were original research articles. Publication years spanned 2011 to 2026, with more than 85% published after 2019, consistent with the rapid expansion of this field in the immunotherapy era. Although the search covered three Chinese-language databases (CNKI, Wanfang, and VIP) with corresponding Chinese search terms, no Chinese-language study met the inclusion criteria of this review: the records retrieved mainly did not meet the criteria (e.g., they focused on diseases other than lung cancer or lacked mechanistic content in the three target domains). Incomplete capture of Chinese-language mechanistic studies is acknowledged as a limitation.

## 3 Results

### 3.1 Soluble metabolites: the chemical language of lung-gut-brain communication

#### 3.1.1 The chemical signaling logic of metabolites

Communication between the gut microbiota and the lung does not rely on direct cell contact. Instead, it operates through diffusible chemical mediators, microbial metabolites, that carry information between organs (9, 21). These molecules are produced by colonic microbiota through fermentation of dietary fiber, metabolism of host-derived substrates such as bile acids, or *de novo* synthesis. They drain into the portal vein via the mesenteric veins, undergo first-pass metabolism in the liver, enter the systemic circulation, and ultimately reach the lung tumor microenvironment (21). This anatomical gut-liver-systemic circulation-lung route forms the structural basis for the remote regulation of lung cancer immune homeostasis by metabolites (**Figure 3**).

**Figure 3:**
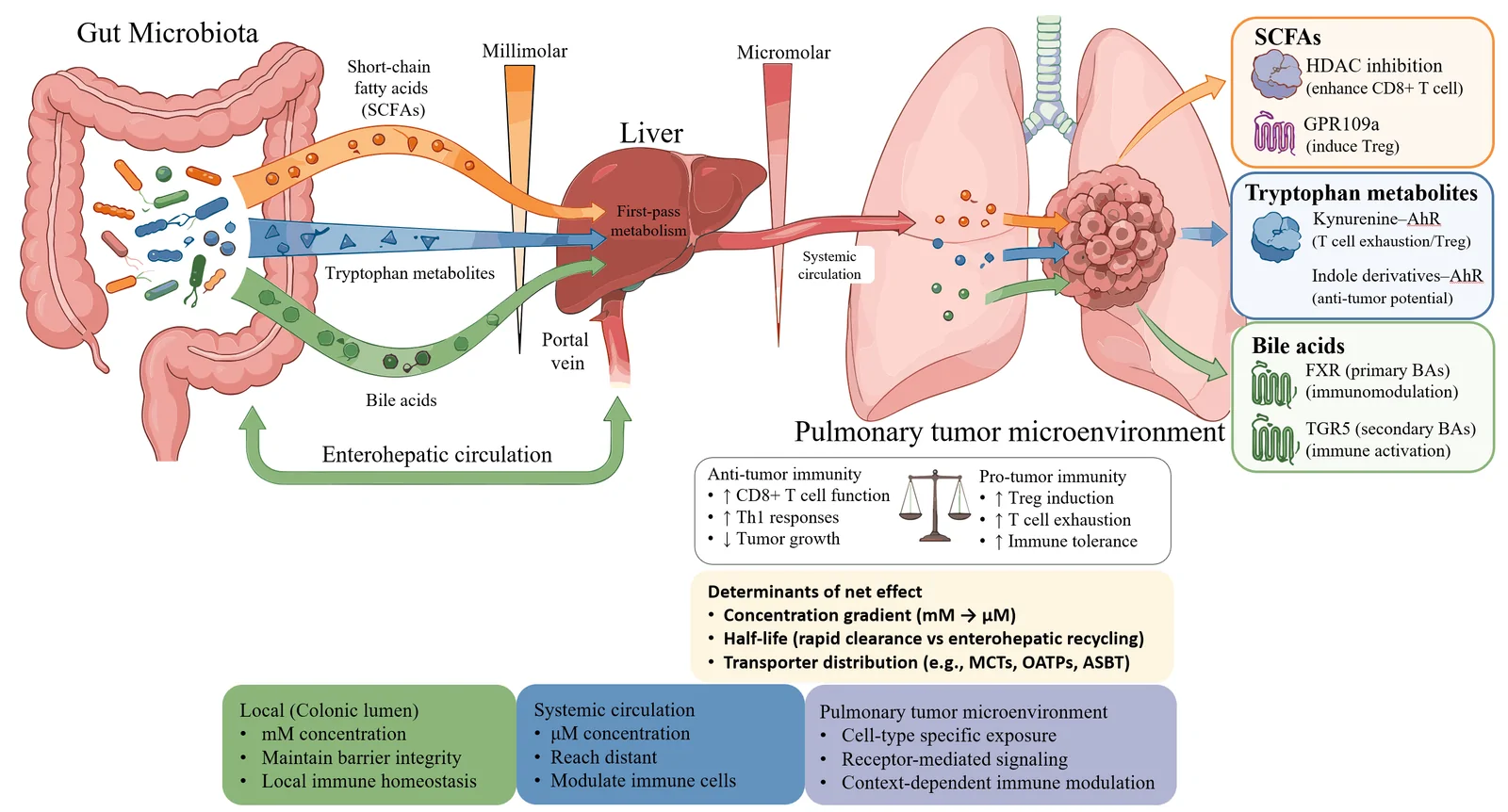
Soluble metabolites regulating the lung tumor microenvironment. SCFAs, tryptophan metabolites, and bile acids produced by gut microbial fermentation enter the portal vein, undergo first-pass hepatic metabolism, and reach the lung tumor microenvironment through the systemic circulation. SCFAs enhance CD8+ T-cell antitumor function through HDAC inhibition while simultaneously inducing regulatory T-cell (Treg) differentiation via GPR109a. Tryptophan metabolites exert bidirectional effects through the aryl hydrocarbon receptor (AhR): the kynurenine pathway drives T-cell exhaustion and immune tolerance, whereas indole derivatives carry antitumor potential. Bile acids mediate differential immune regulation through farnesoid X receptor (FXR; primary bile acids) and Takeda G protein-coupled receptor 5 (TGR5; secondary bile acids). The net immune effect of the three metabolite classes is jointly determined by concentration gradients (from millimolar in the colonic lumen to micromolar in the circulation), molecular half-life, and transporter distribution, yielding context-dependent functional outputs in the local gut, systemic circulation, and lung tumor microenvironment.

Among the three signaling dimensions of the lung-gut-brain axis, soluble metabolites have the densest mechanistic evidence. The effects of SCFAs, tryptophan metabolites, and bile acids have been cross validated in clinical cohorts, animal models, and *in vitro* experiments (18, 22). The operating logic of this chemical signal is defined by three parameters, concentration, half-life, and transporter distribution, each of whose direction of effect is highly context dependent. Concentration determines the radius of effect: millimolar SCFA concentrations in the colonic lumen maintain epithelial barrier integrity, while micromolar concentrations in the systemic circulation are sufficient to activate G protein-coupled receptors (GPR41/GPR43/GPR109A) on immune cell surfaces (9). Yet the same molecule can produce opposing immune effects at different concentrations. Butyrate maintains local immune homeostasis in the colonic lumen but acts on tumor-infiltrating lymphocytes in the circulation (9); sodium propionate selectively induces apoptosis in NSCLC cells with minimal effect on normal lung fibroblasts (23). Half-life determines duration of action: acetate is rapidly cleared from the circulation, whereas deconjugated bile acids, governed by enterohepatic recycling, persist substantially longer in the body (21). Different half-lives mean that the functional output of metabolite signals may vary across distinct treatment windows. During chemotherapy, SCFAs mainly act by preserving the gut barrier and reducing bacterial translocation (11); during immunotherapy, they face a functional antagonism between HDAC inhibition (synergizing with anti-PD-1) and GPR109a signaling (inducing Tregs) (9). Transporter distribution determines tissue-specific exposure: differential expression of monocarboxylate transporters MCT1, MCT4, and MCT5 in the colonic epithelium creates distinct butyrate concentration gradients at different anatomical locations (24); bile acid distribution relies on the apical sodium-dependent bile acid transporter (ASBT/SLC10A2) and organic anion-transporting polypeptide (OATP) families that mediate enterohepatic circulation and systemic distribution (25) Han et al. (26) found that the metabolite profiles of SCLC patients with brain metastases differed significantly from those of non-metastatic patients, further confirming the biological relevance of spatial localization. Taken together, these observations indicate that metabolites do not act as independent molecules but together constitute a chemical signaling system (18). Context-dependence is a fundamental operating principle, not a confounding factor.

#### 3.1.2 Evidence maps for the three core metabolite classes

Short-chain fatty acids, acetate, propionate, and butyrate, are the principal end-products of colonic bacterial fermentation of non-digestible carbohydrates and the most extensively evidenced metabolite class in gut-lung axis research. The three SCFAs show differential receptor-level signaling preferences. Beyond activating GPR109a (also known as niacin receptor 1, NIACR1), butyrate also acts directly as a histone deacetylase (HDAC) inhibitor, producing epigenetic effects that extend beyond membrane receptor signaling through altered chromatin acetylation states. Propionate and acetate preferentially activate GPR41 (free fatty acid receptor 3, FFAR3) and GPR43 (free fatty acid receptor 2, FFAR2), transducing downstream effects through the Gαi/o-calcium signaling cascade (9, 24) Butyrate possesses bidirectional potential: it induces Treg differentiation through GPR109a while simultaneously enhancing CD8+ T-cell effector function through HDAC inhibition (including upregulation of granzyme B and interferon-γ expression), and SCFAs can also drive naive CD4+ T-cell differentiation toward the Th1 direction, cooperatively strengthening antitumor immunity (24).The balance between these two effects depends on concentration: millimolar butyrate in the colonic lumen maintains epithelial barrier integrity and local immune homeostasis, whereas micromolar butyrate in the systemic circulation acts primarily on circulating and tumor-infiltrating immune cells, determining the net immune effect in a given therapeutic context (9).

Beyond receptor-level signaling, the downstream molecular events through which SCFAs shape antitumor immunity deserve attention. At the transcriptional level, butyrate and propionate act as histone deacetylase (HDAC) inhibitors, increasing histone acetylation at effector-gene promoters and thereby enabling the transcription of cytotoxic programs in CD8+ T cells; this epigenetic mechanism extends beyond membrane receptor signaling and represents a major route of SCFA-mediated T-cell regulation (9, 23, 24). At the effector level, SCFA-driven HDAC inhibition upregulates cytotoxic effector molecules such as granzyme B, perforin, and interferon-γ in CD8+ T cells and natural killer cells, and supports the accumulation of tumor-specific and memory T cells, which are associated with durable antitumor immunity (9, 22, 24). At the metabolic level, gut-microbiota-derived SCFAs contribute to the metabolic reprogramming of immune cells. In a Lewis lung carcinoma model, for instance, Qingfei mixture enhanced antitumor immunity partly through the modulation of mTOR signaling and gut-microbiota-derived SCFAs (27), and in patients with NSCLC, metabolic reprogramming associated with the intestinal flora has been linked to the antitumor immune response (17). Taken together, these transcriptional, effector, and metabolic events help explain how micromolar SCFA concentrations in the circulation can exert meaningful effects on tumor-infiltrating lymphocytes (9, 22).

At the pharmacological intervention level, several studies have provided experimental support from different angles for SCFA-mediated enhancement of immunotherapy efficacy. Baicalin enriched Akkermansia and Clostridia_UCG-014, increasing intestinal SCFA production and restoring immunotherapy response in an anti-PD-1 resistance model (28). Qingfei mixture enhanced CD8+ T-cell antitumor function through modulation of mTOR signaling and gut microbiota-derived SCFAs in a Lewis lung carcinoma model (27). Shenling Baizhu Powder remodeled the gut microbiota (enriching Akkermansia, Lactobacillus, Muribaculum, and other beneficial genera), upregulated SCFA levels, and synergized with PD-1 monoclonal antibody to enhance antitumor immunity while alleviating multi-organ immune-related adverse events (29).Yang et al. (11) transplanted feces from immunotherapy responders into tumor-bearing mice with SCFA supplementation, which enhanced intratumoral effector T-cell activity and improved the efficacy of combined chemo-immunotherapy.

At the clinical association level, evidence linking baseline fecal SCFA levels to immunotherapy outcomes is accumulating. In a prospective NSCLC cohort, Yang et al. (11) found that SCFA-producing taxa (Faecalibacterium, Subdoligranulum) were significantly enriched in responders, with gas chromatography-mass spectrometry (GC-MS) quantification confirming elevated butyrate and propionate levels. Ren et al. (30) validated the positive correlation between SCFA levels and immunotherapy response in an independent cohort. At the opposite end of the disease spectrum, cancer cachexia patients exhibit systemic SCFA depletion: Ni et al. (31) used shotgun metagenomic sequencing to identify reduced abundances of Prevotella copri and other species in cachectic patients; Ubachs et al. (32) confirmed decreased fecal SCFA concentrations; and Jeong et al. (33) further validated the impact of cachexia on gut microbiota composition and SCFA metabolism in a Lewis lung carcinoma model, including reduced alpha diversity and significant declines in acetate and butyrate proportions.

The central biological question in tryptophan metabolism concerns the competition of three pathways for the same substrate. Under physiological conditions, the gut microbiota can direct tryptophan toward 5-HT synthesis through tryptophan decarboxylase, or toward indole metabolites (indole-3-acetic acid, indole-3-propionic acid) through tryptophanase. When indoleamine 2,3-dioxygenase (IDO) and tryptophan 2,3-dioxygenase (TDO) are upregulated by tumor or chronic inflammation, metabolic flux shifts: kynurenine production increases while 5-HT and indole synthesis are suppressed through substrate competition. Kynurenine then acts as an endogenous ligand for the aryl hydrocarbon receptor (AhR), driving T-cell exhaustion and Treg induction and forming a key metabolic checkpoint in tumor immune evasion (21, 22). Zhao et al. (18) identified melatonin, indole-3-propionic acid, and skatole as shared differential metabolites in a multi-cancer plasma metabolomic comparison, suggesting that gut microbial tryptophan metabolism plays a common role across multiple malignancies.

Ginseng polysaccharides (GPs) provided experimental validation of this metabolic bifurcation. Huang et al. (34) found that GPs combined with anti-PD-1 monoclonal antibody reshaped the gut microbiota composition, reduced the kynurenine/tryptophan (Kyn/Trp) ratio, increased valeric acid levels, and was accompanied by decreased Foxp3+ Tregs and increased CD8+ effector T cells, demonstrating that rebalancing tryptophan metabolic flux through gut microbiota intervention is a feasible strategy for enhancing immunotherapy efficacy. Sun et al. (35) provided mechanistic evidence from the 5-HT pathway: phenelzine combined with Akkermansia muciniphila enhanced antitumor immunity in NSCLC by modulating the gut microbial metabolic end-product 5-hydroxyindoleacetic acid (5-HIAA). Song et al. (36) found that Bifidobacterium animalis suppressed NSCLC progression through indole-3-acetic acid, suggesting that indole metabolites can exert antitumor effects independently of the kynurenine pathway. Although indole-3-acetic acid also possesses AhR ligand activity, its downstream effect spectrum differs from the classical pro-tolerogenic effects of kynurenine, indicating that the direction of AhR ligand effects depends jointly on ligand type, concentration, and target cell context (21).

The metabolic logic of bile acid signaling is rooted in the enterohepatic circulation, an anatomical-biochemical circuit. Primary bile acids (cholic acid, chenodeoxycholic acid) are synthesized from cholesterol in the liver and secreted into the duodenum. Roughly 95% are reabsorbed in the terminal ileum; the remainder enters the colon, where gut microbial bile salt hydrolases and 7α-dehydroxylase convert them into secondary bile acids (deoxycholic acid, lithocholic acid). Primary and secondary bile acids activate farnesoid X receptor (FXR) and Takeda G protein-coupled receptor 5 (TGR5) with different affinities, producing differential immune and metabolic effects (25). Lee et al. (37) found through targeted metabolomics that taurolithocholic acid was associated with improved immunotherapy survival, could enhance T-cell activation, and its levels correlated closely with the relative abundance of Eubacterium. Xu et al. (10) provided key clinical reverse-genetic validation in an NSCLC cohort: antibiotic exposure disrupted the 7α-dehydroxylation function of the gut microbiota, leading to disordered secondary bile acid metabolism, which in turn significantly weakened chemo-immunotherapy efficacy. Critically, it was not the antibiotics themselves but the resulting disruption of the bile acid metabolic pathway that mediated the loss of efficacy. The mechanism by which the gut microbiota regulates the tumor immune microenvironment through bile acid metabolism has been well validated in NSCLC. Xiao et al. (38), in a study of SCLC liver metastasis, further suggested that this bile acid metabolic pathway may apply broadly to multiple lung cancer subtypes and warrants investigation in larger prospective studies.

#### 3.1.3 From detection to precision intervention

The translation of metabolite research toward clinical application can be divided into three progressive levels: detection, intervention, and precision modulation. At the detection level, baseline fecal butyrate and propionate concentrations can distinguish chemo-immunotherapy responders from non-responders in NSCLC (11); in SCLC, Song et al. (39) and Sun et al. (40) separately confirmed significant differences in microbiota composition and metabolite profiles between long progression-free survival (PFS) and short-PFS groups (11). At the intervention level, the efficacy of direct metabolite supplementation has been validated in animal models (11); ginseng polysaccharides (34) and Shenling Baizhu Powder (29) represent typical paradigms of prebiotic strategies; Huang et al. (41) confirmed the clinical feasibility of fecal microbiota transplantation in a phase 2 study of NSCLC with secondary PD-1 resistance; and Zhou et al. (13) developed a clinic-responder-derived defined microbial consortium (RCom) that correlated positively with immunotherapy response, enhancing anti-PD-1 efficacy by upregulating colonic CD8+ T-cell function and elevating cecal SCFA levels, thereby circumventing the dose-control challenge of single-metabolite supplementation through validation based on clinical responder-derived strains. At the precision modulation level, the framework requires matching the most appropriate intervention strategy to baseline fecal metabolite profiles (SCFA composition, Kyn/Trp ratio, bile acid spectrum) and cachexia status (15)—this framework remains at the proof-of-concept stage, but its core logic has already received substantive support from the multi-level experimental evidence described above.

Soluble microbial metabolites are the core mediators of lung-gut chemical communication. Short-chain fatty acids, tryptophan metabolites, and bile acids produced by the gut microbiota are generated in the colonic lumen, enter the portal vein, undergo hepatic metabolism, enter the systemic circulation, and reach the lung tumor microenvironment via the bloodstream. In lung tissue, SCFAs enhance CD8+ T-cell cytotoxic function through HDAC inhibition while simultaneously inducing Treg differentiation via GPR109a signaling; tryptophan metabolism is skewed toward the kynurenine pathway in the tumor context, driving T-cell exhaustion; and bile acids differentially regulate immune responses through FXR/TGR5 receptors. The net immune effect of the three metabolite classes is jointly determined by concentration gradients, half-life, and tissue distribution, collectively regulating local immune homeostasis in lung cancer.

### 3.2 The metabolic-endocrine axis: enteroendocrine hormones and systemic metabolic reprogramming

#### 3.2.1 EEC endocrine relay: mechanisms and physiological validation

SCFAs and bile acids act both as the chemical diffusion signals described above and as natural ligands for enteroendocrine cells (EECs). GPR41/GPR43 activation stimulates L cells to release GLP-1 and PYY (42), and TGR5 promotes L-cell GLP-1 secretion (43), thereby constituting an endocrine relay pathway distinct from the chemical diffusion mechanism. EECs convert luminal chemical signals into hormonal pulses delivered to distal organs through the systemic circulation (**Figure 4**).

**Figure 4:**
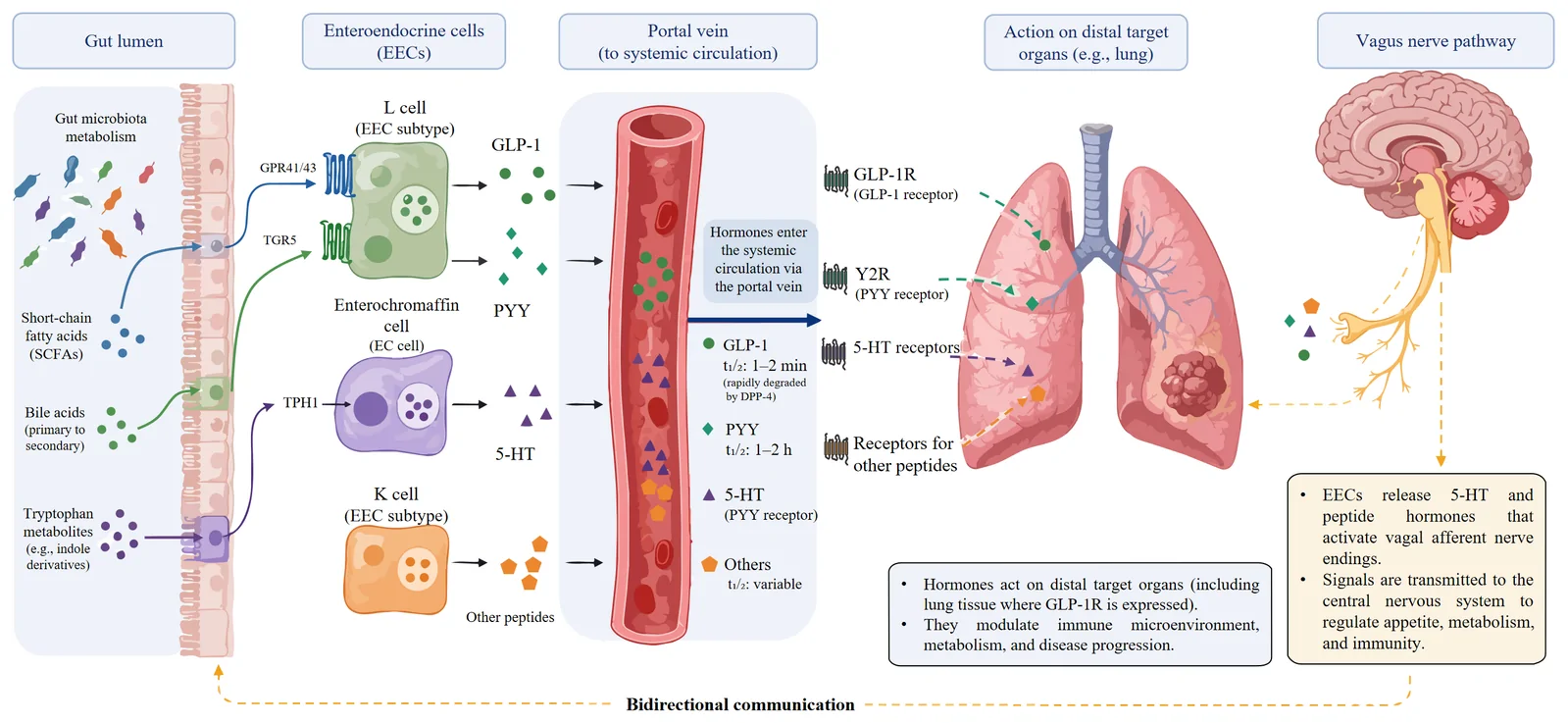
Schematic of the gut–lung metabolic–endocrine signaling axis. Gut microbial metabolites (SCFAs, bile acids, and others) act on enteroendocrine cells (EECs) in the intestinal epithelium, converting luminal chemical signals into hormonal signals for remote regulation. SCFAs activate L cells through GPR41/43 and bile acids through TGR5, triggering secretion of GLP-1 and PYY. Enterochromaffin (EC) cells synthesize 5-HT through the tryptophan hydroxylase-1 (TPH1) pathway. These hormones enter the systemic circulation via the portal vein and act on corresponding receptors expressed in lung tissue, modulating the immune status of the lung tumor microenvironment and disease progression. In addition, bioactive substances released by EECs can activate vagal afferent nerve terminals, forming bidirectional communication with the central nervous system and jointly participating in the integrated regulation of metabolism, appetite, and immunity.

Signal specificity is encoded jointly by EEC subtype distribution along the intestine, hormone half-life, and receptor expression on distal targets (44). A direct signaling connection also exists between EECs and vagal afferent terminals, through which EEC-derived 5-HT and peptide hormones can activate the vagus nerve (45).

Each step of this relay has been validated under normal physiological conditions: SCFA- and bile-acid-stimulated hormone secretion via EEC receptors has been established (44); GLP-1 receptor (GLP-1R) expression has been confirmed in lung tissue (46); and chemotherapy can directly activate EEC secretion of GLP-1, PYY, and 5-HT (47), indicating that EEC activation also occurs in the cancer-treatment context.

#### 3.2.2 The bridging gap in the tumor context

The true gap lies not in the individual steps themselves but in the connections between them: do the relationships established under normal physiological conditions still hold when transposed into the tumor context? Under tumor-bearing conditions, does an equivalent SCFA/bile acid stimulus still elicit an equivalent GLP-1 secretory response—amplified or attenuated? Can circulating GLP-1 reach the lung tumor microenvironment at effective concentrations? Does the signaling connection between EECs and the vagus nerve also exist in NSCLC? Each question corresponds to the extension of a step with existing foundational evidence into the tumor context (44–46), but this extension has yet to be tested. Guo et al. (17) and Yan et al. (14) touched on related issues in conceptual reviews, but did not fill these gaps.

This gap is especially pronounced in cachexia research. The cachexia field has accumulated evidence on microbiota composition and SCFA metabolism, but gut hormones have not been incorporated as measurement endpoints. Ni et al. (31) observed enrichment of Methanobrevibacter smithii in cachectic patients and invoked, in the discussion, the known pathway of methane-stimulated L-cell GLP-1 release and appetite suppression to explain the anorexia phenotype. The logical chain linking microbiota alteration, hormonal mediator, and clinical manifestation is internally consistent, but GLP-1 levels were not measured. The discussion’s invocation amounted to *post hoc* explanation, not causal testing.

#### 3.2.3 Current evidence for key hormones and metabolites

Unlike GLP-1 and PYY, 5-HT involves both microbial metabolism and host endocrine pathways, placing it at a distinct intersection within the endocrine axis. Host EC cells synthesize 5-HT through tryptophan hydroxylase-1 (TPH1), representing endocrine output; tryptamine and indole metabolites produced by gut bacteria through tryptophan decarboxylase and tryptophanase represent microbial metabolic products (48). For the endocrine axis, the core question is whether circulating 5-HT levels in NSCLC patients are regulated by the gut microbiota. However, the existing literature has focused not on host endocrine output but on bacterial tryptophan metabolites. Sun et al. (35) found that phenelzine combined with A. muciniphila enhanced antitumor immunity in NSCLC by modulating gut 5-HIAA. This study targeted the host 5-HT-to-5-HIAA pathway through the monoamine oxidase (MAO) inhibitor phenelzine, rather than bacterially derived tryptophan metabolites. Song et al. (36) found that B. animalis suppressed NSCLC progression through indole-3-acetic acid (IAA), which is produced directly from tryptophan by bacterial tryptophanase without involving the host TPH1 pathway. Both studies provide associational evidence linking microbial tryptophan metabolism to NSCLC immune response, but the measured endpoints were bacterial enzyme products, not host endocrine output.

Secondary bile acids sit directly upstream of the endocrine axis: they promote L-cell GLP-1 secretion through TGR5 (43), giving bile acids the dual role of immunomodulatory metabolite and endocrine signal precursor. Studies have established associations between bile acids and NSCLC immunotherapy outcomes (10, 37, 38), but these studies measured the bile acids themselves; the downstream endocrine output (GLP-1) has not been included as a mediating variable. GLP-1R is expressed in lung tissue, and L-cell GLP-1 secretion is dually regulated by SCFAs (via GPR41/GPR43) and bile acids (via TGR5). It follows that gut microbiota alterations in NSCLC patients should affect the lung immune microenvironment through an SCFA-bile acid-GLP-1 endocrine cascade, but hormonal node levels remain unmeasured.

SCFA regulation of insulin sensitivity has been discussed in reviews (14), and Liu et al. (22) further noted that microbiota-derived SCFAs may drive tumorigenesis through IGF-1 signaling, though this pathway has also not been tested in NSCLC. Evidence from gastric cancer provides a supporting precedent: circulating hormones can promote myeloid-derived suppressor cell (MDSC) expansion and suppress natural killer (NK) cell and CD8+ T-cell function through the glucocorticoid receptor (49), indicating that circulating hormones can remodel the tumor immune microenvironment. If this principle holds in NSCLC, gut-derived hormonal regulation of the lung immune microenvironment would open a new direction for intervention.

In summary, the core pathway of the metabolic-endocrine axis can be outlined as follows: the gut microbiota metabolizes SCFAs and bile acids; these activate L cells through EEC surface receptors (GPR41/GPR43, TGR5), triggering release of GLP-1 and PYY (42–44); hormones enter the systemic circulation via the portal vein and act on distal target tissues, including GLP-1R expressed in lung tissue (46); and EECs also maintain a direct synaptic connection with the vagus nerve (45).

#### 3.2.4 How gut hormones may modulate immune effector cells: testable hypotheses

Direct evidence that GLP-1, PYY, or 5-HT modulate immune effector cells within the lung tumor microenvironment is lacking in NSCLC, but three premises make this question testable rather than speculative. First, GLP-1R is expressed in lung tissue (46). Second, a precedent exists that circulating hormones can remodel the tumor immune microenvironment: in gastric cancer, hormones acting through the glucocorticoid receptor promote myeloid-derived suppressor cell expansion and suppress NK and CD8+ T-cell function (49). Third, host 5-HT metabolism has already been linked to antitumor immunity in NSCLC, since inhibition of the 5-HT-to-5-HIAA pathway with phenelzine enhanced immunotherapy efficacy (35), and chemotherapy itself activates EEC secretion of GLP-1, PYY, and 5-HT (47). On these premises we propose three testable hypotheses: (H1) in NSCLC patients, changes in gut microbiota composition drive changes in circulating GLP-1/PYY/5-HT levels, which correlate with intratumoral CD8+ T-cell infiltration and immunotherapy response, testable in a prospective cohort with paired stool, blood, and tumor samples; (H2) GLP-1R signaling modulates immune effector cells in the lung TME, testable by GLP-1R agonist or antagonist treatment of orthotopic lung cancer models followed by immune phenotyping of the TME; (H3) circulating 5-HT, regulated in part by the gut microbiota, modulates antitumor immunity in NSCLC, testable by TPH1 inhibition or 5-HT receptor blockade in lung cancer models and by measuring circulating 5-HT in patients. Each hypothesis targets a specific unmeasured node of the metabolic–endocrine axis identified above, and each can be tested with existing methods.

### 3.3 Neuroimmune integration: gut-brain-lung signaling converges in the tumor microenvironment

The concept that the gut microbiota remotely regulates the lung immune microenvironment through multiple parallel pathways, the gut-lung axis, has been systematically elaborated in several reviews (2, 8). Research spans from the role of the gut microbiota in lung carcinogenesis (50) and the modulation of immunotherapy efficacy by microbial metabolites (11, 21) to the delivery of immunomodulatory molecules across the gut barrier by microbial extracellular vesicles (4), covering scales from macroecology to molecular carriers. None, however, have addressed the nervous system as a relay mechanism. The neuroimmune axis is the dimension of the lung-gut-brain circuit with substantial conceptual integrative potential yet sparse empirical evidence. But gut microbiota dysbiosis contributing to distant organ disease is not unique to lung cancer. The review by Yan et al. (14) showed that, from neuropsychiatric disorders and metabolic diseases to malignancies, metabolite exchange, epigenetic regulation, and gut-brain axis signaling are shared cross-disease mechanisms. Three neuroanatomical pathways could, in theory, transmit gut-derived signals to pulmonary antitumor immunity: (1) vagal afferent fibres terminating in the nucleus tractus solitarius (NTS), sensing microbial metabolites and translocated bacterial components, with efferent cholinergic signaling modulating peripheral immunity (51); (2) the HPA axis providing an endocrine relay, with microbiota-driven glucocorticoid elevation suppressing T and NK cells (49); and (3) neuroinflammation, driven by chemotherapy, tumor-derived factors, or systemic inflammation, disrupting central-peripheral immune communication through microglial activation and blood-brain barrier (BBB) disruption (52).

#### 3.3.1 The vagus nerve: physical transport and signal sensing

Existing evidence is distributed across animal experiments and human metabolite measurements. At the animal level, Kumar et al. (53) At the animal level, Kumar et al.(52) demonstrated that subdiaphragmatic vagotomy significantly reduced pulmonary bacterial load in a mouse model of interstitial lung disease, providing evidence for the vagus nerve as a physical transport conduit for gut–lung microbial translocation. Two caveats apply. First, this study is currently available only as a bioRxiv preprint and has not yet undergone peer review. Second, it was performed in a non-cancer (interstitial lung disease) model rather than in lung cancer. By the evidence-grading logic used throughout this review, it therefore constitutes cross-context inference on unreviewed data and is coded as preliminary evidence rather than direct NSCLC evidence. At the human level, Zhao et al. (18) detected altered tryptophan metabolites and neuroactive compounds in the plasma of lung cancer patients, signaling molecules that vagal afferent terminals could, in principle, sense.

#### 3.3.2 HPA axis: indirect evidence for an endocrine relay

Evidence comes from parallel studies in other cancer types and clinical associations in lung cancer cachexia. Zhang et al. (49) proposed a microbiota-HPA axis-immunosuppression conceptual framework in gastric cancer. Zhao et al. (18) detected altered neuroactive compounds in lung cancer patient plasma that could be linked to HPA axis output. The most direct evidence comes from lung cancer cachexia: NSCLC cachexia patients harbor a gut microbiome significantly different from that of non-cachectic patients (13, 14), and a colon cancer cachexia model further showed that a microbiota-immune signaling axis drives muscle wasting (16). The cachexia state itself is accompanied by HPA axis activation, and the microbiota differences suggest potential microbial involvement.

#### 3.3.3 Neuroinflammation: brain metastasis and chemotherapy-related cognitive impairment

This axis has the greatest volume of evidence, though with a pronounced cross-cancer gradient in causal clarity. Evidence comes from two clinical scenarios with markedly different causal resolution. The first is the brain metastasis scenario, where brain metastases perturb all three pathways simultaneously through BBB disruption, exogenous glucocorticoid exposure, and systemic inflammation. Jiang et al. (54) found more pronounced gut dysbiosis in NSCLC patients with brain metastases. Han et al. (26) found significantly altered microbiota and metabolite profiles in SCLC patients with brain metastases. Du et al. (55) found distinct microbiota composition in nude mice with leptomeningeal metastases compared with controls. Taken together, these findings indicate an association between neuroinflammation, lung cancer brain metastases, and gut microbiota alterations. In the chemotherapy-related cognitive impairment (CRCI) scenario, the causal chain is clearer: chemotherapy acts as the upstream trigger, driving microglial activation and BBB disruption through systemic inflammation, leading to cognitive decline. A systematic review of cancer treatment-related psychoneurological toxicities has identified microbiota-gut-brain axis (MGBA) association patterns across multiple cancer types, covering fatigue, anxiety, depression, sleep disturbance, and cognitive impairment (47). Mechanistically, these toxicities are transmitted to cognitive impairment through three pathways: microglial activation, oxidative stress, and BBB injury (52, 56). In breast cancer, the evidence chain for these mechanisms is relatively complete, spanning mouse models (57), an RCT (58), and longitudinal studies (59), with multi-level support for the association between post-chemotherapy gut dysbiosis and cognitive impairment (60). In NSCLC, however, the dysbiosis-to-neuroinflammation-to-cognitive-impairment chain has not been tested in its entirety. The tryptophan-kynurenine pathway (34), SCFAs (11, 24), microbial vesicles (61) and microbiota-metabolite-cytokine associations (62) have all focused on immunotherapy efficacy or cancer prevention. A multi-omics analysis of thalidomide for chemotherapy-induced nausea and vomiting in SCLC established only an association between microbiota, metabolites, and antiemetic effects (63). Together, these studies delineate the existence of gut-lung-immune integration, but the neural integration dimension is absent throughout. The FITNESS study (64), which longitudinally collected microbiome and functional disability data in older NSCLC patients, found preliminary correlations between the two. This is the only NSCLC cohort approaching the question, but it did not include neurological measures. In sum, the existing literature sketches the outline of gut-lung-immune connections without touching the neural relay that links the gut to the brain. That relay is precisely the gap that remains to be filled.

## 4 Discussion

This review maps the mechanistic evidence for the lung-gut-brain axis in NSCLC along three lines of inquiry: soluble microbial metabolites, the metabolic-endocrine axis, and neuroimmune integration. The volume of evidence across the three axes shows a pronounced asymmetric distribution. This asymmetry is itself perhaps more informative than the findings of any single pathway: descriptive work linking microbiota composition to immunotherapy outcomes is abundant, but causal mechanism exploration remains heavily concentrated in the chemical dimension, with the endocrine and neural axes nearly blank. Despite the disparity in evidence depth, two cross-axis commonalities deserve attention. First, the immune effects of microbial metabolites and gut-derived hormones depend on concentration, half-life, and tissue distribution; the same molecule (such as butyrate) can produce opposite net effects in different therapeutic contexts (9). Second, the three signaling lines are not isolated from one another: SCFAs can activate L cells to release GLP-1, and a direct synaptic connection exists between EECs and vagal afferent terminals (42, 45). Intervention at any node may produce network-level downstream effects that exceed expectations.

Viewed from the perspective of functional output, the ultimate effects of the three relay lines in the lung can be grouped into two opposing sets of pathways: protective and pathogenic. On the protective side, SCFAs reinforce the epithelial barrier, maintain immune homeostasis through HDAC inhibition, and counter SOX9-driven epithelial-mesenchymal transition (9, 11, 65, 66). On the pathogenic side, dysbiosis triggers pro-inflammatory cytokine release and gut barrier disruption; endotoxin entering the circulation exacerbates pulmonary immune dysregulation (65, 67, 68). The opposition of these two pathway sets suggests that the net immune effect of the lung-gut-brain axis may not be determined by the activity of any single molecule or species, but by the relative dominance of protective versus pro-inflammatory signals. When SCFA-producing bacteria predominate and the barrier is intact, protective pathways may prevail; when dysbiosis occurs, the pro-inflammatory cascade is activated while SCFA signaling declines, potentially forming a self-reinforcing cycle. In ecological terms, this regulatory logic can be understood as tumor-ecosystem modulation: the therapeutic goal is not to improve microbiota composition in a general sense, but to attempt to restore the dominance of protective signals over pro-inflammatory ones. For clinical translation, this points to three operational principles: selecting intervention strategies based on baseline fecal metabolite profiles (SCFA composition, Kyn/Trp ratio, bile acid spectrum) (11, 37); adjusting regulatory targets according to treatment phase (induction, immunotherapy maintenance, resistance progression); and using multi-omics integration (metagenomics, metabolomics, immune repertoire) to explore individualized regimens (13). The differential responses of distinct NSCLC molecular subtypes (epidermal growth factor receptor [EGFR]-mutant, anaplastic lymphoma kinase [ALK]-fusion, etc.) to lung-gut-brain axis modulation also merit investigation as pre-specified stratification variables in future studies.

From a translational perspective, the three axes offer complementary entry points for immunotherapy. Regarding biomarker development, baseline fecal SCFA profiles have distinguished chemo-immunotherapy responders from non-responders in NSCLC (11), the kynurenine/tryptophan ratio has been modified by microbiome-directed interventions (34), and bile acid signatures have been associated with immunotherapy outcomes (10, 37); together with circulating endocrine markers (GLP-1, PYY, 5-HT) that remain to be measured, these constitute a multi-analyte panel whose predictive value for ICI response could be tested prospectively. Regarding ICI responsiveness, the axes map onto distinct resistance mechanisms: SCFA-mediated enhancement of effector T-cell function and the Treg-inducing effects of GPR109a signaling define a concentration-dependent trade-off (9); the kynurenine–AhR axis drives T-cell exhaustion and immune tolerance (21, 22); bile acids modulate antitumor immunity through FXR and TGR5 signaling (25, 69); and the endocrine and neuroimmune relays, though untested in NSCLC, provide mechanistically plausible routes by which host physiology could modulate ICI efficacy. Regarding combination strategies, the most advanced examples are microbial interventions — FMT for patients with secondary PD-1 resistance (41) and in combination with ICI (6), and defined microbial consortia enhancing anti-PD-1 efficacy (13) — while pharmacological candidates include SCFA-enhancing prebiotics, GLP-1 receptor agonists, which are clinically established agents in metabolic disease (70), and monoamine oxidase inhibitors targeting the 5-HT pathway (35). Finally, NSCLC molecular subtypes merit subtype-stratified investigation: EGFR-mutant and ALK-fusion tumors are associated with low response rates to PD-1 pathway blockade (71), whereas KRAS-mutant tumors, particularly those with concurrent TP53 mutations, may derive greater benefit from PD-1 blockade (72); whether these subtypes also differ in their responses to gut-microbiota-directed axis modulation is a testable question that can be addressed by pre-specified stratification in the prospective cohorts proposed in Section 3.1.

Three landmark studies in 2018 (73–75) established the correlation between the gut microbiome and ICI efficacy, and observational studies have since accumulated in large numbers (2, 7, 8, 30). Existing reviews have covered topics from dysbiosis to immunotherapy resistance (2, 8, 17), but all have confined their scope to the chemical dialog of the microbiome-immune interface, without incorporating neuroimmune integration or the metabolic-endocrine axis as independent mechanistic dimensions. In comparison with these works, this review differs in three respects. First, it juxtaposes three signaling pathways and expands the gut-lung axis into the lung-gut-brain axis. Second, it annotates evidence levels at key nodes along each pathway, transforming knowledge gaps from vague conclusions into testable hypotheses. Third, it presents the evidence asymmetry itself as a structural finding, suggesting that future research may concentrate resources on filling the mechanistic gaps in the endocrine and neural dimensions.

### 4.1 A hypothesis-driven pathway model and research agenda

Based on the above evidence, we outline a pathway model annotated with evidence levels at each node (**Figure 2**). The model first distinguishes two roles of the vagus nerve, physical transport conduit and neural signal relay, which should not be conflated. The former transports viable bacteria (pulmonary bacterial load decreases after vagotomy in a non-cancer mouse model; evidence from a non-peer-reviewed preprint (53)), whereas the latter transmits neural signals (vagal afferents sense metabolites and relay through the NTS to the CNS). The two differ in their cargo and in whether the CNS is involved, and they belong to distinct mechanistic levels.

Pathway 1 (physical transport). After gut dysbiosis, viable bacteria can translocate along the vagus nerve as a physical conduit to the lungs. In a mouse model of interstitial lung disease, subdiaphragmatic vagotomy significantly reduced pulmonary bacterial load, suggesting a transport function of the vagus nerve; this evidence derives from a single non-peer-reviewed preprint (bioRxiv) in a non-cancer model and should therefore be regarded as preliminary (53). This pathway bypasses the CNS; bacteria act directly on the lung microenvironment. Whether the translocated microbiota carry immunomodulatory signals and what their actual effect on antitumor immunity may be remains to be tested (hypothetical extrapolation).

Pathway 2 (neuroendocrine signal transduction). Gut microbial metabolites and bacterial components are sensed by vagal afferent terminals, relayed through the NTS to the paraventricular nucleus (PVN), activating the HPA axis and elevating glucocorticoids, which in turn suppress NK cell and CD8+ T-cell function. This chain consists of five steps: (i) vagal afferent sensing of microbial signals (hypothetical extrapolation; no direct evidence in tumor immunology); (ii) NTS-to-PVN central relay (indirect inference: anatomical connection is established (76), untested in tumor immunology); (iii) HPA axis activation leading to glucocorticoid elevation (indirect inference: a framework has been proposed in gastric cancer, untested in lung cancer (49)); (iv) glucocorticoid suppression of NK/CD8+ T cells (indirect inference: the gut microbiome of NSCLC cachexia patients differs significantly from that of non-cachectic patients; a microbiota-immune signaling axis drives muscle wasting in a colon cancer cachexia model (16); (v) net effect on pulmonary antitumor immunity (hypothetical extrapolation; none of the five steps in the complete chain have been validated in an NSCLC model).

Chemotherapy-exacerbated branch. Chemotherapy-induced dysbiosis converges onto both pathways: increasing bacterial translocation (Pathway 1) and amplifying HPA axis activation through heightened systemic inflammation (Pathway 2). In addition, chemotherapy-induced systemic inflammation independently drives microglial activation and BBB disruption leading to CRCI. This pathway has evidence in breast cancer (52, 56, 77) but has not been fully tested in NSCLC.

The primary value of this model lies not in validation, most nodes remain hypothetical, but in its function as a provisional map: it marks which segments of the gut-brain-lung circuit have empirical support and which await exploration. The node-level evidence described above comes almost entirely from indirect inference or from NSCLC gut-lung axis studies that did not measure neural mediating variables (11, 14), indicating that the neuroimmune dimension remains a major knowledge gap. The core tools needed to fill this gap, metabolomics, vagotomy models, neuroimaging, and cognitive assessment, are all mature; they have simply not yet been systematically introduced into the design of NSCLC microbiome studies. Along these lines, several experimental directions may merit priority. First, direct validation of the vagus-HPA-lung immune circuit: the evidence level of each node in Pathway 2 has been annotated, and the chain can be tested in NSCLC-specific models (such as subdiaphragmatic vagotomy combined with orthotopic lung tumor xenografts). Second, the complete SCFA-bile acid-GLP-1 endocrine cascade to the lung immune microenvironment: GLP-1R expression in lung tissue is confirmed (46), but the intermediate nodes have never been measured in full. A prospective cohort simultaneously collecting stool (microbiota and SCFAs) and blood (GLP-1, PYY, 5-HT, and IGF-1 profiles) would test the entire chain in a single design. Given the widespread use of GLP-1 receptor agonists in metabolic disease, the clinical urgency of this gap is considerable. Third, identification of the context-dependent molecular switch of SCFAs: the functional antagonism of butyrate between HDAC inhibition (enhancing CD8+ T cells) and GPR109a signaling (inducing Tregs) (2, 11) may be the key prerequisite for determining whether SCFAs can become a reliable immune adjuvant. None of these paths requires building new methodological platforms; each involves introducing existing tools into the design of NSCLC microbiome research.

### 4.2 Strengths and limitations

The review contributes to several respects.

1. At the framework level, it is, to our knowledge, among the first to integrate the three signaling pathways of soluble metabolites, the metabolic-endocrine axis, and neuroimmune integration into a unified lung-gut-brain axis framework, departing from previous reviews that focused exclusively on the chemical dimension.
2. At the methodological level, it documented a systematic search of seven databases (four English-language and three Chinese-language) following the PRISMA 2020/PRISMA-S reporting guidance, with two authors independently completing study selection, data extraction, and thematic classification, reducing selection bias.
3. At the evidence-handling level, it annotated the evidence level of key nodes in each pathway (direct NSCLC evidence, cross-cancer parallel inference, hypothetical extrapolation), transforming knowledge gaps from vague statements into hypotheses testable node by node.
4. At the integrative level, it identified cross-axis commonalities, including the context-dependence of metabolite effects and the cross-coupling among the three signaling lines, providing a conceptual framework for future mechanistic studies.

Several limitations also apply.

1. All included studies were published in English; although the search covered seven Chinese and English databases, no Chinese-language study met the inclusion criteria, and Chinese-language mechanistic studies may therefore not have been fully captured.
2. Given the high heterogeneity of included studies in design and outcome measures, a narrative synthesis rather than quantitative meta-analysis was used, and effect sizes across pathways could not be pooled.
3. Although the three pathways were discussed separately for narrative clarity, they are biologically highly intertwined, and the interactions and feedback among pathways require further characterization.
4. In the neuroimmune integration and metabolic-endocrine axis dimensions, the number of studies directly addressing core mechanisms is limited. Some nodes in the hypothesis-driven pathway model are based on indirect inference, and the conclusions in these dimensions await validation through more direct evidence.
5. The vagus-nerve physical-transport pathway currently rests on a single non-peer-reviewed preprint performed in an interstitial lung disease mouse model (Section 2.3.1); this node should be regarded as preliminary until peer-reviewed replication in a lung cancer model is available.
6. Because this is a narrative review with a systematic search, formal risk-of-bias and GRADE assessments were not performed. These choices follow from the review’s aim of mapping the maturity of mechanistic evidence rather than pooling effect estimates; consequently, the strength of causal claims is limited relative to a full systematic review, and pathway-level conclusions should be read as provisional until validated by direct NSCLC-specific experiments.

## 5 Conclusion

This review integrates the three signaling pathways of soluble metabolites, the metabolic-endocrine axis, and neuroimmune integration into a unified lung-gut-brain axis framework and provides a systematic mapping of the mechanistic evidence for remote regulation of the lung immune microenvironment by the gut microbiota in NSCLC. The mapping reveals a clear picture: the soluble metabolite pathway is relatively mature, whereas direct evidence for the endocrine and neuroimmune dimensions is extremely scarce. This scarcity is precisely the bottleneck constraining the field’s transition from correlational description to causal mechanism studies. The hypothesis-driven pathway model proposed here offers a node-by-node testable roadmap for this knowledge gap, but its clinical value depends on causal validation through subsequent experiments. As this is a narrative review with a systematic search, the conclusions should be read as a map of evidence maturity rather than as a quantitative synthesis.
