# Correction: The lung-gut-brain axis in non-small-cell lung cancer: a narrative review with a systematic search of three signaling pathways

**DOI:** 10.3389/fimmu.2026.2000931

**Published:** 2026-09-24

**Authors:** Yijun Fan, Lili Du, Qiuping Luo, Tingrui Mei, Aier Zhou, Hangyu Liu, Muhammad Quais Khan, Faxian Gao, Jingsi Dong

**Affiliations:** 1 West China School of Medicine, Sichuan University, Chengdu, China; 2 Department of Endocrinology and Metabolism, The Fourth Affiliated Hospital of Harbin Medical University, Harbin, China; 3 Out-patient Department, West China Hospital, Sichuan University, Chengdu, China; 4 Department of Thoracic Surgery, West China Hospital, Sichuan University, Chengdu, China; 5 Lung Cancer Center/Lung Cancer Institute, West China Hospital, Sichuan University, Chengdu, China

**Keywords:** gut microbiota, metabolic-endocrine axis, neuroimmune integration, non-small-cell lung cancer, soluble metabolites

Affiliation “Lung Cancer Center/Lung Cancer Institute, West China Hospital, Sichuan University, Chengdu, China” was omitted from the paper. This affiliation has now been added for authors “Tingrui Mei, Muhammad Quais Khan, Faxian Gao, and Jingsi Dong”.

The original version of this article has been updated.

