# Supplementary material for: The lung-gut-brain axis in non-small-cell lung cancer: a narrative review with a systematic search of three signaling pathways
Source: Front Immunol. 2026 Sep 11;17:1932225. doi: 10.3389/fimmu.2026.1932225 (PMC13647171; doi:10.3389/fimmu.2026.1932225)
Supplement: Supplementary file 1 [file DataSheet1.docx]

# Appendix 1. Search Strategies and Results

## 1. Search strategies

Seven Chinese and English databases were searched systematically: PubMed, EMBASE, Web of Science, Cochrane Library, CNKI, Wanfang Data, and VIP, from database inception to June 2026, restricted to English and Chinese. Eight complementary strategies (S1–S8) were designed around the three signaling pathways of the lung–gut–brain axis in lung cancer. Queries were built using PubMed syntax as the baseline and adapted to the syntax of each database; corresponding Chinese search terms were used in the Chinese databases.

**S1 — Lung–gut–brain axis ∩ lung cancer (broad)**

- **PubMed:** (“lung-gut-brain axis” OR “lung-gut axis” OR “gut-lung-brain axis” OR “gut-lung axis”) AND (“lung cancer” OR “lung tumor” OR “lung carcinoma” OR “NSCLC” OR “SCLC” OR “small cell lung cancer” OR “non-small cell lung cancer”)
- **EMBASE (adapted):** (‘lung-gut-brain axis’ OR ‘lung-gut axis’ OR ‘gut-lung-brain axis’ OR ‘gut-lung axis’) AND (‘lung cancer’/exp OR ‘lung tumor’/exp OR ‘lung carcinoma’/exp OR ‘non-small cell lung cancer’/exp OR ‘small cell lung cancer’/exp)
- **Web of Science (adapted):** TS=((“lung-gut-brain axis” OR “lung-gut axis” OR “gut-lung-brain axis” OR “gut-lung axis”) AND (“lung cancer” OR “lung tumor” OR “lung carcinoma” OR “NSCLC” OR “SCLC” OR “small cell lung cancer” OR “non-small cell lung cancer”))
- **Cochrane Library (adapted):** (“lung-gut-brain axis” OR “lung-gut axis” OR “gut-lung-brain axis” OR “gut-lung axis”) AND (“lung cancer” OR “lung tumor” OR “lung carcinoma” OR “NSCLC” OR “SCLC” OR “small cell lung cancer” OR “non-small cell lung cancer”) (text words :ti,ab,kw, combined with MeSH terms where applicable)
- **CNKI / Wanfang / VIP (Chinese terms):** (“肺-肠-脑轴” OR “肺肠轴” OR “肠-肺-脑轴” OR “肠肺轴”) AND (“肺癌” OR “肺肿瘤” OR “非小细胞肺癌” OR “小细胞肺癌”)

**S2 — Gut microbiota ∩ lung cancer ∩ neuroimmune**

- **PubMed:** ((“gut microbiota” OR “gut microbiome” OR “intestinal microbiota”) AND (“lung cancer” OR “lung tumor” OR “NSCLC” OR “SCLC”)) AND (“neuroimmune” OR “neuro-immune” OR “vagus nerve” OR “neuroendocrine” OR “HPA axis” OR “hypothalamic-pituitary” OR “neuroinflammation” OR “neural regulation”)
- **EMBASE (adapted):** ((‘gut microbiota’/exp OR ‘gut microbiome’ OR ‘intestinal microbiota’) AND (‘lung cancer’/exp OR ‘lung tumor’/exp OR ‘non-small cell lung cancer’/exp OR ‘small cell lung cancer’/exp)) AND (‘neuroimmunology’/exp OR ‘neuroimmune’ OR ‘vagus nerve’/exp OR ‘neuroendocrine’ OR ‘hypothalamus hypophysis adrenal axis’ OR ‘neuroinflammation’ OR ‘nerve regulation’)
- **Web of Science (adapted):** TS=(((“gut microbiota” OR “gut microbiome” OR “intestinal microbiota”) AND (“lung cancer” OR “lung tumor” OR “NSCLC” OR “SCLC”)) AND (“neuroimmune” OR “neuro-immune” OR “vagus nerve” OR “neuroendocrine” OR “HPA axis” OR “hypothalamic-pituitary” OR “neuroinflammation” OR “neural regulation”))
- **Cochrane Library (adapted):** ((“gut microbiota” OR “gut microbiome” OR “intestinal microbiota”) AND (“lung cancer” OR “lung tumor” OR “NSCLC” OR “SCLC”)) AND (“neuroimmune” OR “neuro-immune” OR “vagus nerve” OR “neuroendocrine” OR “HPA axis” OR “hypothalamic-pituitary” OR “neuroinflammation” OR “neural regulation”) (text words :ti,ab,kw, combined with MeSH terms where applicable)
- **CNKI / Wanfang / VIP (Chinese terms):** ((“肠道菌群” OR “肠道微生物群” OR “肠道微生态” OR “肠菌”) AND (“肺癌” OR “非小细胞肺癌” OR “小细胞肺癌”)) AND (“神经免疫” OR “迷走神经” OR “神经内分泌” OR “下丘脑-垂体-肾上腺轴” OR “HPA轴” OR “神经炎症”)

**S3 — Gut microbiota ∩ lung cancer ∩ soluble metabolites**

- **PubMed:** (“gut microbiota” OR “gut microbiome”) AND (“lung cancer” OR “lung tumor” OR “NSCLC” OR “SCLC”) AND (“short chain fatty acids” OR “SCFA” OR “metabolites” OR “metabolomics” OR “butyrate” OR “propionate” OR “acetate” OR “tryptophan” OR “TMAO” OR “bile acids” OR “soluble mediator”)
- **EMBASE (adapted):** (‘gut microbiota’/exp OR ‘gut microbiome’) AND (‘lung cancer’/exp OR ‘lung tumor’/exp OR ‘non-small cell lung cancer’/exp OR ‘small cell lung cancer’/exp) AND (‘short chain fatty acid’/exp OR ‘SCFA’ OR ‘metabolite’/exp OR ‘metabolomics’/exp OR ‘butyric acid’ OR ‘propionic acid’ OR ‘acetic acid’ OR ‘tryptophan’ OR ‘trimethylamine n-oxide’ OR ‘bile acid’/exp)
- **Web of Science (adapted):** TS=((“gut microbiota” OR “gut microbiome”) AND (“lung cancer” OR “lung tumor” OR “NSCLC” OR “SCLC”) AND (“short chain fatty acids” OR “SCFA” OR “metabolites” OR “metabolomics” OR “butyrate” OR “propionate” OR “acetate” OR “tryptophan” OR “TMAO” OR “bile acids” OR “soluble mediator”))
- **Cochrane Library (adapted):** (“gut microbiota” OR “gut microbiome”) AND (“lung cancer” OR “lung tumor” OR “NSCLC” OR “SCLC”) AND (“short chain fatty acids” OR “SCFA” OR “metabolites” OR “metabolomics” OR “butyrate” OR “propionate” OR “acetate” OR “tryptophan” OR “TMAO” OR “bile acids” OR “soluble mediator”) (text words :ti,ab,kw, combined with MeSH terms where applicable)
- **CNKI / Wanfang / VIP (Chinese terms):** (“肠道菌群” OR “肠道微生物群” OR “肠道微生态”) AND (“肺癌” OR “非小细胞肺癌” OR “小细胞肺癌”) AND (“短链脂肪酸” OR “代谢物” OR “代谢组学” OR “丁酸” OR “丙酸” OR “乙酸” OR “色氨酸” OR “氧化三甲胺” OR “TMAO” OR “胆汁酸”)

**S4 — Lung cancer ∩ gut–brain axis ∩ cognitive impairment**

- **PubMed:** (“lung cancer” OR “NSCLC” OR “SCLC”) AND (“gut-brain axis” OR “microbiota-gut-brain” OR “brain-gut axis”) AND (“cognitive impairment” OR “chemobrain” OR “chemo brain” OR “chemotherapy-related cognitive impairment” OR “CRCI” OR “depression” OR “anxiety”)
- **EMBASE (adapted):** (‘lung cancer’/exp OR ‘non-small cell lung cancer’/exp OR ‘small cell lung cancer’/exp) AND (‘brain gut axis’ OR ‘microbiota-gut-brain’ OR ‘gut brain axis’) AND (‘cognitive defect’/exp OR ‘chemobrain’ OR ‘chemotherapy related cognitive impairment’ OR ‘depression’/exp OR ‘anxiety’/exp)
- **Web of Science (adapted):** TS=((“lung cancer” OR “NSCLC” OR “SCLC”) AND (“gut-brain axis” OR “microbiota-gut-brain” OR “brain-gut axis”) AND (“cognitive impairment” OR “chemobrain” OR “chemo brain” OR “chemotherapy-related cognitive impairment” OR “CRCI” OR “depression” OR “anxiety”))
- **Cochrane Library (adapted):** (“lung cancer” OR “NSCLC” OR “SCLC”) AND (“gut-brain axis” OR “microbiota-gut-brain” OR “brain-gut axis”) AND (“cognitive impairment” OR “chemobrain” OR “chemo brain” OR “chemotherapy-related cognitive impairment” OR “CRCI” OR “depression” OR “anxiety”) (text words :ti,ab,kw, combined with MeSH terms where applicable)
- **CNKI / Wanfang / VIP (Chinese terms):** (“肺癌” OR “非小细胞肺癌” OR “小细胞肺癌”) AND (“肠-脑轴” OR “菌群-肠-脑轴” OR “脑-肠轴”) AND (“认知障碍” OR “化疗脑” OR “化疗相关认知障碍” OR “抑郁” OR “焦虑”)

**S5 — SCLC ∩ microbiome (broad)**

- **PubMed:** (“small cell lung cancer” OR “SCLC”) AND (“gut microbiota” OR “gut microbiome” OR “intestinal microbiota” OR “microbiome”)
- **EMBASE (adapted):** (‘small cell lung cancer’/exp OR ‘SCLC’) AND (‘gut microbiota’/exp OR ‘gut microbiome’ OR ‘intestinal microbiota’ OR ‘microbiome’/exp)
- **Web of Science (adapted):** TS=((“small cell lung cancer” OR “SCLC”) AND (“gut microbiota” OR “gut microbiome” OR “intestinal microbiota” OR “microbiome”))
- **Cochrane Library (adapted):** (“small cell lung cancer” OR “SCLC”) AND (“gut microbiota” OR “gut microbiome” OR “intestinal microbiota” OR “microbiome”) (text words :ti,ab,kw, combined with MeSH terms where applicable)
- **CNKI / Wanfang / VIP (Chinese terms):** (“小细胞肺癌” OR “SCLC”) AND (“肠道菌群” OR “肠道微生物群” OR “肠道微生态” OR “微生物组”)

**S6 — Lung–gut axis ∩ neuroendocrine hormones**

- **PubMed:** (“lung-gut axis” OR “gut-lung axis”) AND (“neuroendocrine” OR “HPA axis” OR “cortisol” OR “serotonin” OR “5-HT” OR “GLP-1” OR “enteric nervous” OR “vagus”)
- **EMBASE (adapted):** (‘lung gut axis’ OR ‘gut lung axis’) AND (‘neuroendocrinology’/exp OR ‘hypothalamus hypophysis adrenal axis’ OR ‘hydrocortisone’/exp OR ‘serotonin’/exp OR ‘5-hydroxytryptamine’ OR ‘glucagon like peptide 1’/exp OR ‘enteric nervous system’/exp OR ‘nervus vagus’/exp)
- **Web of Science (adapted):** TS=((“lung-gut axis” OR “gut-lung axis”) AND (“neuroendocrine” OR “HPA axis” OR “cortisol” OR “serotonin” OR “5-HT” OR “GLP-1” OR “enteric nervous” OR “vagus”))
- **Cochrane Library (adapted):** (“lung-gut axis” OR “gut-lung axis”) AND (“neuroendocrine” OR “HPA axis” OR “cortisol” OR “serotonin” OR “5-HT” OR “GLP-1” OR “enteric nervous” OR “vagus”) (text words :ti,ab,kw, combined with MeSH terms where applicable)
- **CNKI / Wanfang / VIP (Chinese terms):** (“肺肠轴” OR “肠肺轴”) AND (“神经内分泌” OR “下丘脑-垂体-肾上腺轴” OR “皮质醇” OR “5-羟色胺” OR “血清素” OR “GLP-1” OR “肠神经系统” OR “迷走神经”)

**S7 — Lung–gut–brain axis (broad, all contexts)**

- **PubMed:** (“lung-gut-brain” OR “gut-brain-lung” OR “brain-lung axis” OR “lung-brain axis”)
- **EMBASE (adapted):** (‘lung gut brain’ OR ‘gut brain lung’ OR ‘brain lung axis’ OR ‘lung brain axis’)
- **Web of Science (adapted):** TS=((“lung-gut-brain” OR “gut-brain-lung” OR “brain-lung axis” OR “lung-brain axis”))
- **Cochrane Library (adapted):** (“lung-gut-brain” OR “gut-brain-lung” OR “brain-lung axis” OR “lung-brain axis”) (text words :ti,ab,kw, combined with MeSH terms where applicable)
- **CNKI / Wanfang / VIP (Chinese terms):** (“肺-肠-脑轴” OR “肠-肺-脑轴” OR “脑-肺轴” OR “肺-脑轴”)

**S8 — SCLC ∩ tumor microenvironment ∩ gut microbiota**

- **PubMed:** (“small cell lung cancer” OR “SCLC”) AND (“immune microenvironment” OR “tumor microenvironment” OR “TME”) AND (“gut microbiota” OR “gut microbiome” OR “intestinal microbiota”)
- **EMBASE (adapted):** (‘small cell lung cancer’/exp OR ‘SCLC’) AND (‘tumor microenvironment’/exp OR ‘immune microenvironment’ OR ‘TME’) AND (‘gut microbiota’/exp OR ‘gut microbiome’ OR ‘intestinal microbiota’)
- **Web of Science (adapted):** TS=((“small cell lung cancer” OR “SCLC”) AND (“immune microenvironment” OR “tumor microenvironment” OR “TME”) AND (“gut microbiota” OR “gut microbiome” OR “intestinal microbiota”))
- **Cochrane Library (adapted):** (“small cell lung cancer” OR “SCLC”) AND (“immune microenvironment” OR “tumor microenvironment” OR “TME”) AND (“gut microbiota” OR “gut microbiome” OR “intestinal microbiota”) (text words :ti,ab,kw, combined with MeSH terms where applicable)
- **CNKI / Wanfang / VIP (Chinese terms):** (“小细胞肺癌” OR “SCLC”) AND (“免疫微环境” OR “肿瘤微环境”) AND (“肠道菌群” OR “肠道微生物群” OR “肠道微生态”)

## 2. Search results

### 2.1 Hit counts per strategy

| Strategy | Thematic dimension | PubMed hits | Other six databases (combined) | Total across all seven databases |
| --- | --- | --- | --- | --- |
| S1 | Lung–gut–brain axis ∩ lung cancer (broad) | 88 | 44 | 132 |
| S2 | Gut microbiota ∩ lung cancer ∩ neuroimmune | 4 | 4 | 8 |
| S3 | Gut microbiota ∩ lung cancer ∩ soluble metabolites | 151 | 69 | 220 |
| S4 | Lung cancer ∩ gut–brain axis ∩ cognitive impairment | 5 | 7 | 12 |
| S5 | SCLC ∩ microbiome (broad) | 398 | 122 | 520 |
| S6 | Lung–gut axis ∩ neuroendocrine hormones | 18 | 17 | 35 |
| S7 | Lung–gut–brain axis (broad, all contexts) | 158 | 68 | 226 |
| S8 | SCLC ∩ tumor microenvironment ∩ gut microbiota | 25 | 37 | 62 |
| **Total** |  | **847** | **368** | **1,215** |

### 2.2 Per-database hit counts (before deduplication)

| Database | Language | Records returned |
| --- | --- | --- |
| PubMed | English | 847 |
| EMBASE | English | 179 |
| Web of Science | English | 151 |
| Cochrane Library | English | 38 |
| CNKI | Chinese | 0 |
| Wanfang Data | Chinese | 0 |
| VIP | Chinese | 0 |
| **Total** |  | **1,215** |

Note: CNKI, Wanfang Data, and VIP each returned 0 records. EMBASE, Web of Science, and Cochrane Library returned 368 records in total (179 + 151 + 38).

### 2.3 Chinese-language search results

The records retrieved from the three Chinese-language databases (CNKI, Wanfang Data, and VIP) **mainly did not meet the inclusion criteria of this review** (e.g., they focused on non-lung-cancer diseases or lacked mechanistic content in the three target domains), rather than being duplicates of English-language publications; consequently, no Chinese-language study was included, and all 168 included studies are English-language publications (consistent with Section 1.10 of the manuscript).
