# Supplementary material for: The lung-gut-brain axis in non-small-cell lung cancer: a narrative review with a systematic search of three signaling pathways
Source: Front Immunol. 2026 Sep 11;17:1932225. doi: 10.3389/fimmu.2026.1932225 (PMC13647171; doi:10.3389/fimmu.2026.1932225)
Supplement: Supplementary file 2 [file DataSheet3.docx]

**Appendix 3. Quality Summary Table**

**Table A3(a). Distribution of the 168 included studies by evidence setting**

| **Evidence setting** | **No. of studies** |
| --- | --- |
| Human | 80 |
| Review | 54 |
| Human + animal | 14 |
| Animal | 11 |
| Animal + in vitro | 4 |
| Human + animal + in vitro | 4 |
| In vitro | 1 |
| Total | 168 |

**Table A3(b). Distribution of the 168 included studies by study-design category**

| **Study-design category** | **No. of studies** |
| --- | --- |
| Clinical observational / bioinformatic / protocol | 72 |
| Review / meta-analysis / bibliometric | 55 |
| Animal model | 31 |
| Clinical trial (RCT / phase I–III) | 8 |
| In vitro | 1 |
| Other | 1 |
| Total | 168 |

**Table A3(c). Distribution of the 168 included studies by cancer type**

| **Cancer type** | **No. of studies** |
| --- | --- |
| NSCLC | 91 |
| NSCLC + SCLC (lung cancer) | 36 |
| Pan-cancer (lung included) | 23 |
| NSCLC + other | 7 |
| SCLC | 5 |
| Non-lung cancer (supporting evidence) | 4 |
| Non-cancer mechanistic (incl. interstitial lung disease model) | 2 |
| Total | 168 |

**Table A3(d). Per-theme breakdown of evidence setting (human vs. animal/in vitro vs. review)**

| **Thematic axis** | **Human** | **Animal / in vitro** | **Review** | **Total** |
| --- | --- | --- | --- | --- |
| Neuroimmune integration | 89 | 13 | 51 | 153 |
| Soluble metabolites | 79 | 15 | 44 | 138 |
| Metabolic–endocrine axis | 61 | 11 | 33 | 105 |

Note: a study could be assigned to more than one thematic axis; the counts in Table A3(d) therefore reflect the breadth of thematic coverage (Section 1.9) and sum to more than 168.

Interpretation: The soluble-metabolites axis is supported predominantly by clinical cohorts (79 human studies) with corroborating animal and in vitro experiments, whereas the metabolic–endocrine and neuroimmune axes rely more heavily on reviews and on cross-context inference. This heterogeneity of evidence quality is reflected in the node-level evidence grading of Figure 4 and in the correspondingly cautious wording of the conclusions for the endocrine and neuroimmune dimensions.
